# Molecular genetics in laboratory medicine: from foundations to the future of precision diagnostics

**DOI:** 10.1515/almed-2026-0087

**Published:** 2026-07-07

**Authors:** Giuseppe Lippi, Giorgia Beffagna

**Affiliations:** Section of Clinical Biochemistry and School of Medicine, University of Verona, Verona, Italy; Division of Cardiology, Department of Medicine, University of Verona, Verona, Italy

**Keywords:** laboratory medicine, molecular genetics, molecular biology, genetics

Molecular genetics has substantially evolved from a highly specialized research discipline into an essential component of modern laboratory medicine [1]. Few areas in diagnostics have transformed clinical laboratories as deeply as molecular genetics, not only from a technological perspective, but also in terms of professional expertise, laboratory organization, clinical interpretation, and interaction with stakeholders (i.e., clinicians and patients). Molecular diagnostics now virtually permeates every branch of science and medicine, including oncology, hematology, infectious diseases, inherited disorders, transplantation medicine, reproductive medicine, and pharmacogenomics. This evolution has radically changed the role of clinical laboratories, which is no longer confined to the production of analytical results, but has become an active player in clinical decision-making and personalized patient management.

Molecular genetics may be generally defined as the discipline investigating the structure, function, regulation, and alterations of genes at the molecular level. Within laboratory medicine, this field encompasses several interconnected domains, including genomics, transcriptomics, epigenetics, pharmacogenetics/pharmacogenomics, molecular pathology, molecular microbiology, and cytogenetics (Table 1) [2]. In brief, genomics focuses on the analysis of DNA sequences and genomic organization, while transcriptomics assesses RNA expression profiles and their relationship with cellular function and disease. Epigenetics explores mechanisms that regulate gene expression irrespective of DNA sequence alterations, especially DNA methylation and chromatin remodeling. Pharmacogenetics assesses the influence of genetic variability on drug metabolism and therapeutic response, while molecular pathology integrates molecular alterations with disease pathways and tissue pathology. Molecular microbiology has become essential for the timely and accurate identification of pathogens and for characterizing antimicrobial resistance. Finally, cytogenetics has become a cornerstone of diagnostic laboratory practice, especially for evaluation of hematologic malignancies, constitutional chromosomal disorders, and prenatal conditions. In particular, the introduction of non-invasive prenatal testing (NIPT), expanded carrier screening programs, and high-throughput genomic sequencing has enhanced diagnostic accuracy and broadened the scope of detectable conditions, transforming prenatal risk assessment, diagnostic algorithms, and reproductive counseling strategies [3].

**Table 1:** Molecular genetics may be broadly defined as the discipline investigating the structure, function, regulation, and alterations of genes at the molecular level. Within laboratory medicine, this field encompasses several interconnected domains, each contributing unique diagnostic and therapeutic insights to clinical practice.

Subdiscipline	Definition	Primary focus in laboratory medicine
Genomics	Comprehensive analysis of whole genomes, including sequence variation, structural variation, and genomic architecture	Genome-wide variant detection, gene mapping, structural variant analysis, clinical genomic interpretation
Transcriptomics	Analysis of RNA expression and its relationship to cellular state and disease	Gene expression profiling, RNA sequencing (bulk and single-cell), biomarker discovery
Epigenetics	Study of heritable gene regulation mechanisms not involving DNA sequence changes	DNA methylation profiling, epigenetic signatures in cancer and disease stratification
Pharmacogenetics/pharmacogenomics	Study of genetic variation influencing drug metabolism, efficacy, and toxicity	Genotype-guided drug selection, dosing optimization, prediction of adverse drug reactions
Molecular pathology	Integration of molecular alterations with histopathology for disease classification and prognosis	Tumor molecular profiling, tissue-based NGS testing, genotype–phenotype correlation
Molecular microbiology	Molecular-based detection and characterization of infectious agents and resistance mechanisms	Pathogen identification, antimicrobial resistance profiling, molecular syndromic testing, metagenomics
Cytogenetics	Study of chromosomal structure and numerical/structural abnormalities	Karyotyping, chromosomal microarrays, diagnosis of hematologic and congenital disorders

NGS, next generation sequencing.

The extraordinary development of molecular genetics in clinical laboratories over the last four decades has been driven by successive technological revolutions. The introduction of polymerase chain reaction (PCR) has been a crucial catalyst, enabling the amplification of nucleic acids with sensitivity and specificity previously unimaginable, becoming rapidly integrated into routine diagnostics and changing the approach to severe pathologies like infectious diseases, thrombophilia, inherited disorders and hematologic cancers [4]. The completion of the Human Genome Project (HGP) subsequently accelerated the transition toward large-scale genomic analysis, leading the way to the foundation of precision medicine. However, the real turning point for clinical laboratories has been the advent of next-generation sequencing (NGS) technologies, which transformed molecular diagnostics from targeted single-gene analysis into broad and comprehensive genomic profiling [5]. In contemporary clinical laboratories, NGS is no longer restricted to specialized academic centers, but has progressively entered routine diagnostics. Several laboratories are now capable to perform targeted gene panel analyses and, where clinically indicated, more comprehensive genomic investigations, including whole-exome and whole-genome sequencing. This technological evolution has enormously expanded diagnostic capabilities, especially in areas such as oncology, where molecular profiling is now essential for classification, prognostic assessment, evaluation of minimal residual disease, and monitoring of treatments [6].

The emergence of individualized cancer vaccines based on tumor neoantigen profiling is among the most promising developments [7]. These approaches, which combine NGS with advanced bioinformatics and immunological modeling, represent one of the most paradigmatic examples of “translational” laboratory medicine. Tumor-specific genomic alterations can be identified through molecular analysis and then used to generate personalized vaccines that stimulate patient-specific immune responses. Although still mostly investigational, early studies in certain types of cancer, such as melanoma and pancreatic malignancies, have shown promising results, suggesting that molecular laboratories may soon become directly involved not only in diagnosis but also in the design of personalized therapeutic strategies [7].

Contextually, pharmacogenomics is gradually reshaping routine laboratory practice [8]. Genetic polymorphisms affecting drug-metabolizing enzymes, transport proteins, and therapeutic targets are increasingly influencing clinical decision-making across multiple medical disciplines. Clinical laboratories are hence transitioning from isolated pharmacogenetic assays toward comprehensive pharmacogenomic profiling integrated within electronic medical records and clinical decision-support systems.

Laboratory automation has also deeply influenced the integration of molecular genetics into modern laboratory workflows [9]. High-throughput molecular testing would simply not be feasible without extensive automation of extraction procedures, liquid handling, amplification systems, sequencing workflows, and bioinformatics pipelines. Automation has become indispensable for maintaining acceptable turnaround times, analytical reproducibility, traceability, and quality assurance in laboratories processing thousands of molecular tests per day. The concept of total laboratory automation (TLA), initially developed for clinical chemistry, hematology and hemostasis, is progressively extending into molecular diagnostics, especially in large centralized laboratories [10].

From an operational perspective, laboratory automation clearly improves standardization, traceability and efficiency of workflows throughout the total testing process [11]. Reducing manual steps and enforcing more uniform procedures helps to limit analytical variability and several well-known sources of preanalytical and analytical errors. High-throughput, integrated platforms also enable laboratories to handle increasing test volumes while maintaining rapid turnaround times. Molecular diagnostics of infectious disease are a paradigmatic example, since the timely and accurate detection of pathogens directly affects clinical decisions.

Nevertheless, it should be emphasized that automation does not eliminate the intrinsic analytical and interpretative complexity of molecular diagnostics. The preanalytical phase remains crucial and requires accurate governance to ensure sample quality and nucleic acid integrity [12]. Even when workflows are highly standardized, key steps such as assay validation, result interpretation, variant classification, and assessment of biological and clinical relevance still rely on expert judgment. Quality assurance continues to depend on professional oversight, especially for evaluating discrepant or unexpected test results that automated systems cannot fully contextualize. Automation should hence be considered a valuable adjunct to laboratory practice, enhancing analytical workflows without replacing the critical interpretative expertise of experienced laboratory professionals [13]. One of the major challenges facing modern laboratory medicine is not analytical generation of molecular data, but rather their accurate interpretation. The volume of genomic information currently generated by high-throughput technologies is huge, and often exceeds the current biological understanding. Distinguishing clinically relevant variants from benign polymorphisms or variants of uncertain significance (VUS) remains a complex and evolving framework [14].

The growing complexity of molecular findings also highlights the importance of appropriate genetic counseling before and after testing. Genomic investigations frequently generate results that extend beyond the initial diagnostic question, including not only VUS, but also incidental findings and information with potential implications for family members. Consequently, effective communication of molecular results has become an essential component of patient-centered laboratory medicine. Close collaboration among laboratory professionals, clinical geneticists, genetic counselors, and treating physicians is increasingly necessary to ensure that genomic information is interpreted, communicated, and used appropriately in clinical practice.

The evolution of molecular genetics has also been accelerated by the rapid development of artificial intelligence (AI) and machine learning (ML) applications [15]. AI-based systems are increasingly used for variant classification, genomic pattern recognition, digital pathology, multi-omics integration, and predictive modeling. In principle, these technologies may significantly enhance analytical efficiency and support the interpretation of highly complex datasets. ML algorithms are already contributing to neoantigen prediction for personalized cancer vaccines and to the integration of genomic and clinical data in precision oncology [16]. Despite the reasonable enthusiasm, caution remains essential. AI systems are entirely dependent on quality and representativeness of the data on which they are trained. Limited interpretability and the potential for systematic bias continue to represent significant drawbacks, especially when these systems are integrated into clinical decision-making pathways. Clinical laboratories have hence a central responsibility to validate AI-driven tools before implementation [15].

The increasing emphasis on precision medicine has also catalyzed the rapid commercialization of novel molecular assays, whose clinical utility remains largely debated. Multi-cancer early detection (MCED) tests based on circulating tumor DNA are among the most debated examples. These assays, which aim to detect multiple malignancies through methylation signatures and advanced computational algorithms, are technologically fascinating, but their effective translation into routine clinical practice seems rather premature. Recent analyses and debates have highlighted several unresolved issues, including limited sensitivity for early-stage cancers, a clinically relevant number of false-positive cases, uncertain cost-effectiveness, and the potential to generate false reassurance in patients [17]. This last aspect merits particular focus. A diagnostic test characterized by inadequate sensitivity may paradoxically discourage patients from undergoing validated screening or diagnostic procedures (e.g., mammography, colonoscopy, or fecal occult blood testing) [18]. In this context, the indiscriminate promotion of molecular technologies risks to undermine decades of evidence-based preventive medicine. The history of laboratory diagnostics repeatedly demonstrates that technological sophistication alone does not necessarily translate into tangible clinical benefits. Accordingly, the future of molecular genetics in laboratory medicine should not be driven exclusively by technological enthusiasm, but requires a balanced integration of innovation, scientific rigor, clinical responsibility, and interpretative expertise.

**Figure 1:**
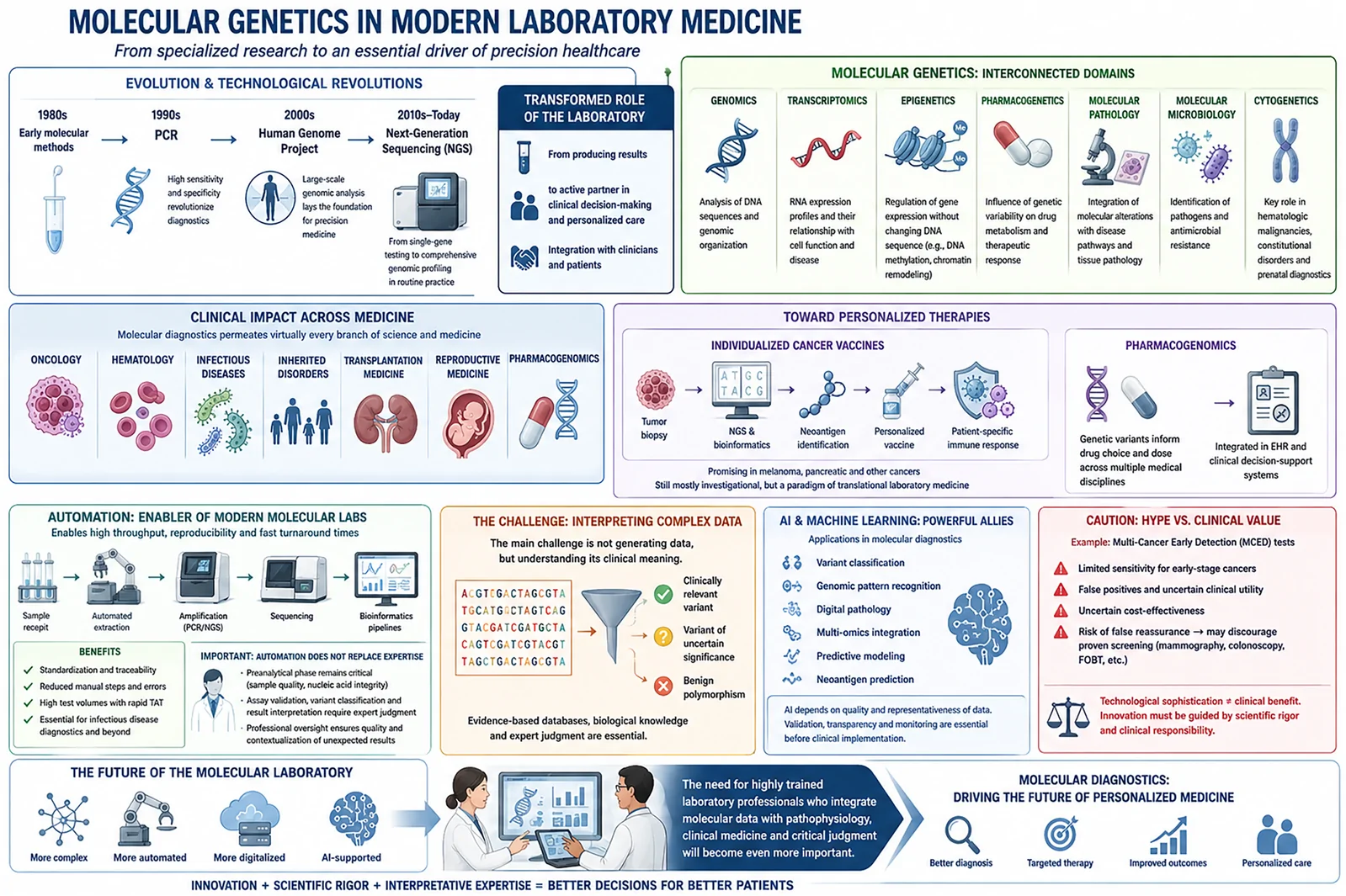
Molecular genetics in laboratory medicine: from foundations to the future of precision diagnostics.

The molecular laboratory of the future will undoubtedly become more complex, automated, digitalized, and increasingly AI-supported. Nevertheless, the role of highly trained laboratory professionals will remain central, as the interpretation of molecular findings continues to require the integration of genomic data with pathophysiological mechanisms, clinical context, and expert judgment (Figure 1).
